# Post-radiotherapy recurrence of conventional oral squamous cell carcinoma showing sarcomatoid components: an immunohistochemical study

**DOI:** 10.4322/acr.2020.219

**Published:** 2020-12-08

**Authors:** Túlio Morandin Ferrisse, Audrey Foster Lefort Rocha, Maria Letícia de Almeida Lança, Heitor Albergoni Silveira, Luciana Yamamoto Almeida, Andreia Bufalino, Jorge Esquiche León

**Affiliations:** 1 Universidade Estadual de São Paulo (UNESP), Faculdade de Odontologia de Araraquara, Departamento de Diagnóstico e Cirurgia, Medicina Oral, Araraquara, SP, Brasil; 2 Universidade de São Paulo (USP), Faculdade de Odontologia de Riberão Preto, Departamento de Estomatologia, Saúde Coletiva e Odontologia Legal, Patologia Oral, Ribeirão Preto, SP, Brasil

**Keywords:** Squamous cell carcinoma, Squamous Cell Carcinoma of Head and Neck, immunohistochemistry, radiotherapy

## Abstract

Spindle cell squamous cell carcinoma (SpSCC) is a rare biphasic malignant neoplasm, uncommonly affecting the oral cavity. The SpSCC diagnosis is difficult, especially when it exhibits inconspicuous morphology, inadequate tissue sampling, or association with an exuberant inflammatory reaction. Post-radiotherapy recurrent SpSCC occurring at the same site of conventional SCC is a rare phenomenon. A 59-year-old man was complained of “painful injury on the tongue” with 20 days of duration. He reported smoking and alcohol consumption. Medical history revealed conventional SCC on the tongue treated with surgery and radiotherapy 10 years ago. Intraoral examination showed a polypoid lesion with ulcerated areas, measuring 3 cm in diameter, on the tongue and floor of the mouth, at the same site of previous conventional SCC. The microscopical analysis showed small foci of carcinomatous component admixed with an exuberant inflammatory reaction. Immunohistochemistry highlighted the sarcomatoid component. Both malignant components were positive for EMA, CD138, p40 (deltaNp63), p63, and p53. Moreover, CK AE1/AE3 evidenced the carcinomatous component, whereas vimentin stained the sarcomatoid component. The Ki-67 was >10%. The current case emphasizes the importance of immunohistochemistry in the differential diagnosis of SpSCC from mimics and documents a rare complication of Ionizing Radiation.

## INTRODUCTION

Squamous cell carcinoma (SCC) accounts for about 90% of the oral and oropharyngeal cancers. Oral cavity SCC (OSCC) is the most common malignancy of the head and neck region.^1^ The tobacco smoking and alcohol consumption are the main etiological factors of the OSCC.^2^ In the last decades, human papillomavirus (HPV) has emerged as a major etiologic factor for oropharyngeal SCC (OPSCC) and on the lips, the ultraviolet radiation plays a central role in the carcinogenesis.^2^ ^-^ ^4^ The OSCC includes several histopathological variants, including verrucous, basaloid, adenoid, spindle cell, adenosquamous and undifferentiated. The understanding of its microscopic peculiarities is fundamental for correct diagnosis and consequently adequate treatment.^5^

Spindle cell SCC (SpSCC) of the head and neck region is a rare, biphasic neoplasm with aggressive behavior.^6^ The main sites of occurrence are the upper aerodigestive tract, larynx, and hypopharynx; nevertheless, in the oral cavity, the occurrence is rare, accounting for less than 1% of all SCCs.^7^ The clinical presentation of SpSCC usually vary from exophytic, polypoid mass with an ulcerated surface to an infiltrative ulcer and the histopathological characteristics exhibit a dysplastic epithelium with foci of infiltration and connective tissue stroma containing numerous spindle-shaped cells, many of them round to oval in shape with eosinophilic and vacuolated cytoplasm, nuclear hyperchromatism and atypical mitoses.^8^ ^,^ ^9^ Interestingly, SpSCC after radiotherapy treatment in some conventional SCC patients have been reported.^10^ Thus, a detailed clinicopathological analysis of similar cases is encouraged better to understand their pathogenesis, treatment, and prognosis.^11^

## CASE REPORT

**Figure 1:**
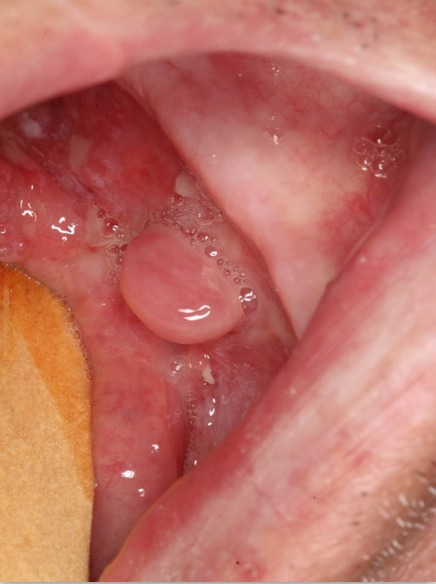
Clinical features of recurrent SpSCC after radiotherapy at the same site of previous conventional well-differentiated SCC. Polypoid nodular lesion on the floor of the mouth and tongue, surrounded by erythematous areas.

A 59-year-old male Caucasian patient was referred with the main complaint of “wound in the tongue” is lasting 20 days. On the clinical examination, a painful, exophytic, polypoid lesion was observed with approximately 3 cm in diameter associated with ulcerative areas on the floor of the mouth with extension to the adjacent tongue (Figure 1).

**Figure 2:**
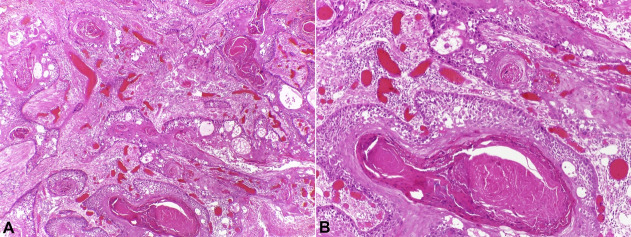
Histopathological features of oral SCC: neoplastic epithelium with conventional pattern (Original magnification, A, x10; B, x20) (H&E stain).

On the extraoral examination, nothing of note was identified. According to the patient, the lesion started 30 days ago, and it increased in size. The medical history was remarkable for a previous diagnosis of well-differentiated SCC in this same region 10 years ago (Figure 2), clinically presented as an ulcerated lesion, being the patient staged as T3N0M0.

**Figure 3:**
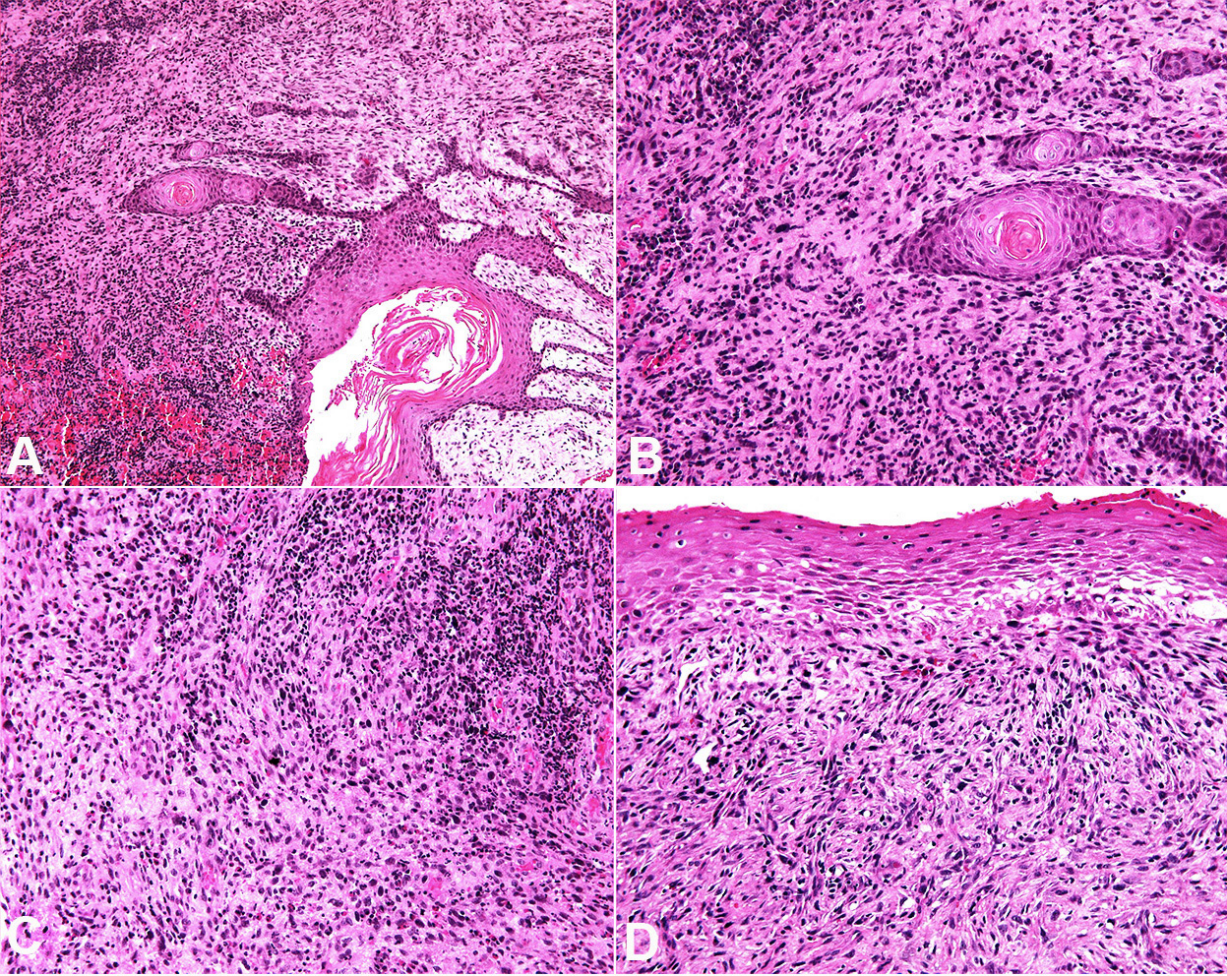
Histopathological features of SpSCC: Biphasic tumor consisting of infiltrating neoplastic epithelium in close relationship to spindle cells (H&E, A, x10; B, x20). Dysplastic epithelium and overt pleomorphism of the spindle cells in the sarcomatoid component (H&E, C, x20; D, x20).

The treatment consisted of surgery and conventional external beam radiotherapy with 70 Gy in 35 sessions. An incisional biopsy was performed, and the histopathological analysis revealed a biphasic tumor consisting of a carcinomatous component evidenced by dysplastic and infiltrative epithelium, focal formation of keratin pearls and an intense inflammatory cell infiltrate permeating a sarcomatoid component (Figure 3).

**Figure 4:**
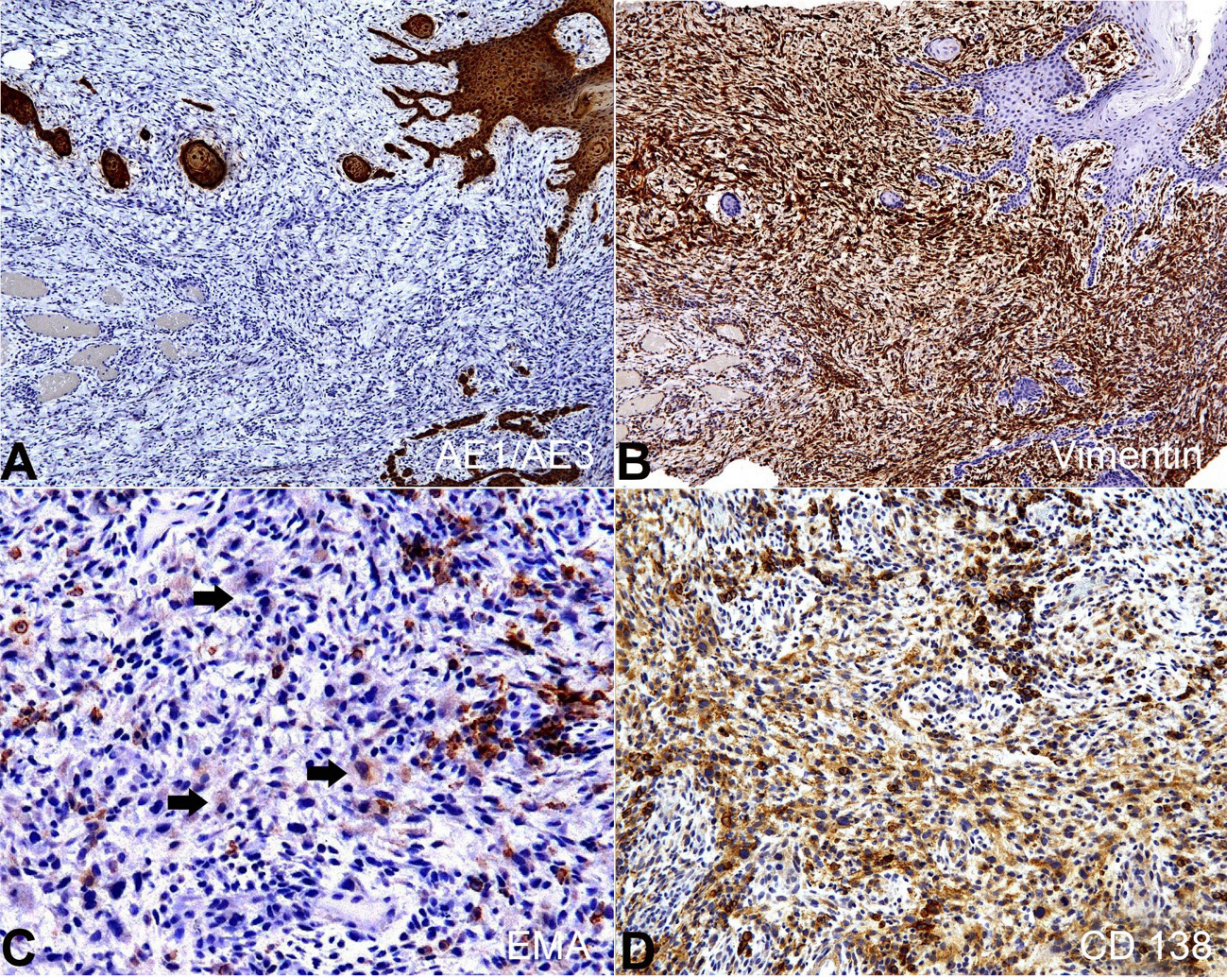
Immunohistochemical analysis of the SpSCC on consecutive serial tissue sections. **A –** Positive reaction for CK AE1/AE3 in the carcinomatous component; **B –** Vimentin highlighted the sarcomatoid component; **C –** EMA expression was weak and detected scarce sarcomatoid cells (arrows); **D –** CD138 evidenced both carcinomatous and sarcomatoid components (A and B x10, C x40 and D x20).

**Figure 5:**
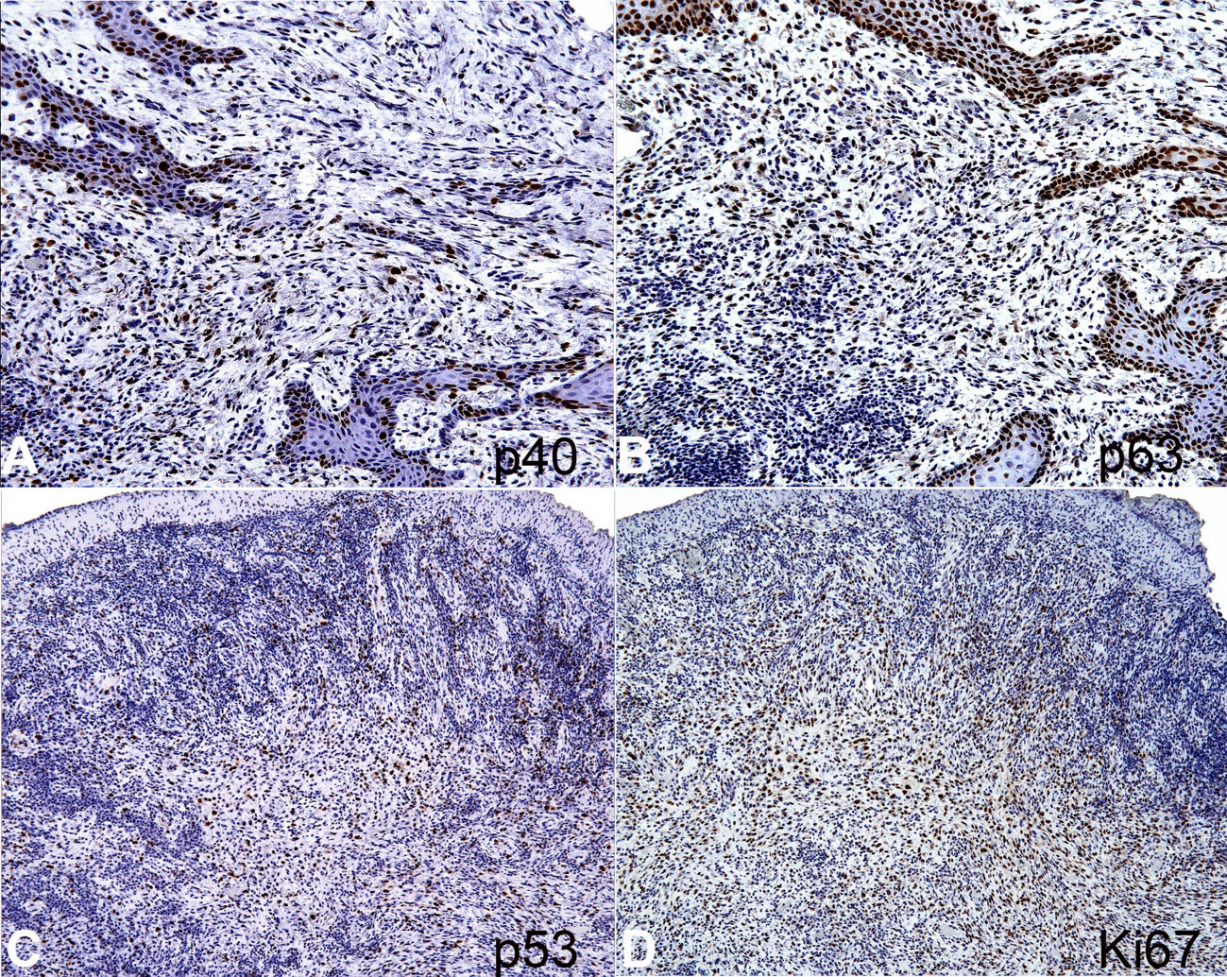
Immunohistochemical analysis of the SpSCC on consecutive serial tissue sections. **A –** Positive reaction for p40 (deltaNp63) in both sarcomatoid and carcinomatous components; as well as in **B –** positivity for p63; **C –** The sarcomatoid component showing positive nuclear staining for p53; and **D –** Ki-67 (>10%) (A and B x20, C and D x10).

The immunohistochemical analysis (Figure 4 and 5) showed positivity for cytokeratin AE1/AE3 highlighting the carcinomatous component, while that vimentin strongly stained the sarcomatoid component. Moreover, EMA, CD138, p40 (deltaNp63), p63, and p53 highlighted both carcinomatous and sarcomatoid components. Of them, only the EMA expression was weak and focal in the sarcomatoid component. S100 protein was negative. The Ki-67 labeling index was >10%. The final diagnosis was SpSCC, and the patient was referred to the oncologist. The patient was submitted to tumor resection and complementary radiotherapy with a total dose of 70 Gy even as the first radiotherapy 10 years ago. After 1-year of follow-up, no sign of recurrence or alteration was observed.

## DISCUSSION

SpSCCs of the head and neck region are rare variants of SCC, representing less than 3% of all head and neck SCCs. The most frequently involved sites in the oral cavity are the lower lip, tongue, and gingiva.^6^ Microscopically, SpSCC is characterized by the presence of two distinct morphological components, carcinomatous and sarcomatoid or spindle cell proliferation, with both components of epithelial origin.^8^ Post-radiotherapy SpSCC is a rare complication. Interestingly, the current case is the third SpSCC originated after surgical resection and radiotherapy of conventional SCC, with both tumors being diagnosed in the same location, at the head and neck region. Taking into consideration that radiotherapy has beneficial effects in the SCC treatment, the development of SCC after radiotherapy is uncommon and deserves special attention to better understand its pathogenesis, treatment, and prognosis.^12^

A systematic review conducted by Brown et al.^13^ in OSCC shows no difference in local recurrence between patients who underwent surgical treatment alone and surgical treatment with postoperative radiotherapy. On the other hand, overall survival seems to be relatively lesser in those patients receiving surgical treatment with postoperative radiotherapy.^13^ Therefore, these results indicate the need for more randomized clinical trials and possible changes in cancer treatment protocols, aiming to improve the quality of life of these patients. Additionally, according to Cahan et al.^14^ study, some tumor types may originate due to exposure to different types of radiation. It is not possible to determine from histopathological examination whether a tumor occurring in a radiation field has been induced by radiation.^14^ ^,^ ^15^ However there are in the scientific literature many report cases of malignant neoplasms, either SCCs or sarcomas, which appeared after treatment with radiotherapy.^7^ ^,^ ^10^ ^,^ ^11^ ^,^ ^16^ ^,^ ^17^

The cause of tumor development in the radiation field may be explained for dose-response effects and the tumor type.^18^ For example, the Colleman study^19^ noted that the induction of sarcomas requires higher doses, especially in thyroid and breast, since lower doses may induce leukemia. For SCC, scientific researches demonstrating the exact amount of dose for carcinogenic induction were not found; however, for SpSCC cases arising after radiotherapy treatment from conventional SCC, the amount of radiation used appears to be sufficient to induce carcinogenesis.^19^ A total radiation dose may vary between 50-70 Gy for SCC in the head and neck region.^20^

According to Baker et al.^21^ study, fibrosis, and vascular changes induced by radiotherapy may be responsible for the development of secondary malignant neoplasms. The radiation can induce chromosome aberrations such as asymmetrical rearrangements that may be observed at the first mitosis in tumor cells, and normal-cells.^22^ Radiation may also induce high levels of micronuclei standing acentric fragments or whole chromosome loss at anaphase.^23^ Thus, further studies of the tumor microenvironment and cell pathways can clarify the possible mechanisms involved in cases of recurrence and neoplasms induced by radiation.

The nonneoplastic effects of radiation on mucosal tissues are dependent on the type of tissue irradiated. The most typical alterations occur in the lamina propria, submucosa, and deep soft tissues of the mouth, pharynx, larynx, and genitalia, which reveal fibrosis and lack or paucity of inflammatory exudate, followed by fibrinous exudate, atypical fibroblasts, and necrosis. Noteworthy, the radiation-induced mesenchymal stromal cell alterations can mimic a malignant neoplasm, either carcinoma or sarcoma.^24^ ^-^ ^26^ Thus, strict clinicopathological correlation, supported by immunohistochemistry, is necessary to achieve a correct diagnosis.

We also conducted a literature review considering recurrence in cases of SpSCC in the head and neck region and other information such as demographic data, primary tumor, location, treatment, and time of recurrence.^7^ ^,^ ^9^ ^,^ ^10^ ^,^ ^27^ ^-^ ^34^ In most cases, the location of recurrence and the initial site of involvement were the same. The initial treatment most commonly used was a combination of surgery and radiotherapy, and surgery alone was often associated with recurrence. The time of recurrence ranged from 2 months to 11 years. Table 1 shows the clinicopathological features of primary conventional SCC and recurrence as SpSCC, and Table 2 shows the clinicopathological features of primary SpSCC and recurrence as SpSCC. It is worth noting that twenty cases of recurrent SpSCC were not included in the tables due to lack of clinicopathological data.^35^ ^-^ ^37^ According to our literature review, there are forty-four cases of recurrent SpSCC in the head and neck region, of which only nine cases originated from conventional SCC. The present study relates to the tenth case.

**Table 1:** Clinicopathological features of primary conventional SCC and recurrence as SpSCC in the head and neck region

Study	Age (y)/Sex	Location	Tx	Recurrence	Site of recurrence	Primary Tumor	Tx	Time to recurrence (y)
Takata et al.^27^	50/M	Gingiva	S+Rad	SpSCC	Oropharynx	SCC	S+Chm	18
	83/F	Buccal mucosa	Rad	SpSCC	Buccal mucosa	SCC	S+Rad+Chm	5
	71/F	Tongue	Rad	SpSCC	Gingiva	SCC	S	2
	76/M	Gingiva/FoM	S+Rad	SpSCC	Tongue	SCC	S	11
Minami et al.^28^	58/F	Esophagus	Rad	SpSCC	Oropharynx	SCC	S	11
Kinra et al.^10^	56/M	Larynx	S+Rad	SpSCC	Oropharynx	SCC	S	3
Oktay et al.^7^	55/F	Tongue	S+Rad	SpSCC	Tongue	SCC	S+Rad	8
Manickam et al.^29^	62/M	Larynx	S+Rad	SpSCC	Larynx	SCC	S	3
Okuyama et al.^30^	62/F	Tongue	Surg	SpSCC	Tongue	SCC	S	4
Index case	49/M	Tongue	S+Rad	SpSCC	FoM/Tongue	SCC	S+Rad	10

Legend: Chm: chemotherapy; F: female; FoM: floor of the mouth; M: male; S: surgery; SCC: squamous cell carcinoma; Rad: radiotherapy; SpSCC: spindle cell squamous cell carcinoma; Tx: treatment; y: years.

**Table 2:** Clinicopathological features of primary SpSCC and recurrence as SpSCC in the head and neck region

**Study**	**Age(y)/sex**	**Ethnicity**	**Location**	**Tx**	**Recurrence**	**Site of recurrence**	**Primary tumor**	**Tx**	**Time to recurrence (yr)**
	N/A	N/A	Tongue	Rad	SpSCC	Tongue	SpSCC	N/A	N/A
	N/A	N/A	Tongue/FoM	Rad	SpSCC	Tongue/FoM	SpSCC	N/A	N/A
	N/A	N/A	Retromolar trigone	Rad	SpSCC	Retromolar Trigone	SpSCC	N/A	N/A
	N/A	N/A	Retromolar trigone Palate	Rad	SpSCC	Retromolar trigone/Palate	SpSCC	N/A	N/A
Leventon and Evans^31^	N/A	N/A	Lower lip	Rad	SpSCC	Lower lip	SpSCC	N/A	N/A
	N/A	N/A	Buccal mucosa	Rad	SpSCC	Buccal mucosa	SpSCC	N/A	N/A
	N/A	N/A	Buccal mucosa	Rad	SpSCC	Buccal mucosa	SpSCC	N/A	N/A
	N/A	N/A	Lip	Rad	SpSCC	Lip	SpSCC	N/A	N/A
	N/A	N/A	Oropharynx	Rad	SpSCC	Oropharynx	SpSCC	N/A	N/A
	76/M	N/A	Gingiva	Chm	SpSCC	Gingiva	SpSCC	N/A	0.2
	75/F	N/A	Tongue	Rad	SpSCC	Tongue	SpSCC	N/A	0.5
	49/M	N/A	FoM	S+Chm	SpSCC	FoM	SpSCC	N/A	0.2
	59/M	N/A	Tongue	S	SpSCC	Tongue	SpSCC	N/A	0.5
	47/M	N/A	Oropharynx	N/A	SpSCC	Oropharynx	SpSCC	N/A	0.5
	42/M	N/A	Oropharynx	S+Rad	SpSCC	Oropharynx	SpSCC	N/A	4.0
	44/M	N/A	Tongue	Surg	SpSCC	Tongue	SpSCC	N/A	1.9
	42/M	N/A	Palate	S+Rad	SpSCC	Palate	SpSCC	N/A	0.7
	75/M	N/A	Buccal mucosa	S	SpSCC	Buccal mucosa	SpSCC	N/A	1.7
Su et al.^32^	52/M	N/A	Buccal mucosa	S+Rad	SpSCC	Buccal mucosa	SpSCC	N/A	0.6
	32/M	N/A	Tongue	S	SpSCC	Tongue	SpSCC	N/A	0.2
	51/M	N/A	Buccal mucosa	S	SpSCC	Buccal mucosa	SpSCC	N/A	2.2
	42/M	N/A	Lip	S+Chm	SpSCC	Lip	SpSCC	N/A	2.7
	59/M	N/A	Gingiva	S	SpSCC	Gingiva	SpSCC	N/A	1.1
	52/M	N/A	Tongue	S+Rad+Ch	SpSCC	Tongue	SpSCC	N/A	1.7
	51/M	N/A	Tongue	Surg	SpSCC	Tongue	SpSCC	N/A	0.2
	46/M	N/A	Lip	S+Rad	SpSCC	Lip	SpSCC	N/A	0.7
	67/M	N/A	Buccal mucosa	S+Rad	SpSCC	Buccal mucosa	SpCC	N/A	0.5
	60/M	N/A	Tongue	S+Rad	SpSCC	Tongue	SpSCC	Surg	3.7
	63/M	N/A	Tongue	S+Rad	SpSCC	Tongue	SpSCC	Surg	1.7
Iqbal et al.^33^	N/A	N/A	Maxilla	S+Rad	SpSCC	Maxilla	SpSCC	Surg	N/A
	N/A	N/A	Maxilla	S+Rad	SpSCC	Maxilla	SpSCC	Surg	N/A
	65/M	N/A	Hypopharynx	S+Rad	SpSCC	Hypopharynx	SpSCC	Surg	4.1
	63/M	N/A	Hypopharynx	S+Rad	SpSCC	Hypopharynx	SpSCC	Surg	1.8
Ohba et al.^34^	72/F	Asian	Buccal mucosa	S+Rad+Ch	SpSCC	Submandibular region	SpSCC	Ch+Rad	1.0
Al-Bayaty and Balkaran^9^	73/F	Black	Gingiva/Mandible	Surg	SpSCC	Gingiva/Mandible	SpSCC	N/A	0.5

Legend: Chm: chemotherapy; F: female; FoM: floor of the mouth; M: male; N/A: not available; Rad: radiotherapy; S: surgery; SpSCC: spindle cell squamous cell carcinoma; Tx: treatment; y: years.

The histopathological diagnosis of SpSCC in the head and neck region is often difficult due to embryologically and anatomically complex area, small sample sizes of biopsy, and often inflammatory cell infiltrate admixed with malignant cells. Moreover, because SpSCC may present four histopathological patterns, including monomorphic, pleomorphic, biphasic, and myxoid, the diagnosis is challenging. In addition, the microscopic distinction of SpSCC from other malignant mesenchymal spindle cell neoplasms may require aid from immunohistochemistry.^38^ The correct understanding of these histopathological aspects is fundamental to achieve the correct diagnosis. In the present study, the sarcomatoid component of the SpSCC was obscured by an intense inflammatory cell infiltrate.

However, after immunohistochemical analysis, the spindle cells revealed positivity for EMA, CD138, p40 (deltaNp63), p63 and p53, with significant proliferative index (Ki-67, >10%). Our findings are in agreement with other reported SpSCC cases.^39^ ^,^ ^40^

## CONCLUSION

Post-radiotherapy recurrent SpSCC occurring at the same site of well-differentiated SCC is a rare phenomenon, usually related to poor prognosis. Herein we report the fourth case in the head and neck region. The histopathological diagnosis can be challenging, because, in some cases, the immunohistochemical analysis is necessary for the correct diagnosis. Further studies assessing recurrent SpSCC after radiotherapy are encouraged better to understand their pathogenesis, treatment, and prognosis.
